# The rate of the 6174delT founder Jewish mutation in *BRCA2* in patients with non-colonic gastrointestinal tract tumours in Israel

**DOI:** 10.1054/bjoc.2000.1605

**Published:** 2001-02

**Authors:** A Figer, L Irmin, R Geva, D Flex, J Sulkes, A Sulkes, E Friedman

**Affiliations:** Institute of Oncology; Epidemiology Unit, Rabin Medical Center (Belinson Campus), Petach Tikva; the Susanne Levy Gertner Oncogenetics Unit Chaim Sheba Medical Center, Tel-Hashomer, 52621, and the Sackler Faculty of Medicine, Tel-Aviv University, Tel-Aviv, Israel

**Keywords:** BRCA 2, Jewish founder mutation, non-colon cancer, inherited predisposition to cancer

## Abstract

Inherited predisposition occurs in 5–10% of all gastrointestinal (GI) cancer patients, but with the exception of colorectal cancer (CRC), the genes involved in conferring genetic susceptibility remain largely unknown. Indirect evidence indicates that germline mutations in *BRCA2* might be associated with an increased risk for various GI malignancies. A single mutation (6174delT) occurs in the *BRCA2* gene in high-risk breast ovarian cancer families of Jewish Ashkenazi origin, in about 1% of the general Ashkenazi population, and rarely in non-Ashkenazi Jews. In order to assess the contribution of this germline mutation to non-CRC GI cancer in Jewish Israeli patients, we tested 70 unselected, consecutive Jewish Ashkenazi patients with gastrointestinal malignancies for this mutation by PCR amplification and modified restriction enzyme digests. Patients' age range was 38–90 years (mean 65.8±11.8 years). The most common malignancies were gastric cancer (*n* = 35) and exocrine pancreatic cancer (*n* = 23). Overall, 6 mutation carriers were detected: 3/23 (13%) of the patients with pancreatic cancer, 2/35 (5.7%) of patients with gastric cancer and 1/4 (25%) of patients with bile duct cancer. The 8.6% mutation carrier rate among patients is a rate significantly higher than that of the general Ashkenazi population (1.16%*P* = 0.0002). We conclude that the rate of the predominant Jewish *BRCA2* mutation in patients with gastric and pancreatic cancer significantly differ from that of the general population of the same ethnic origin. Thus, *BRCA2* mutations probably contribute to gastrointestinal tumorigenesis other then colon cancer, and the surveillance scheme for mutation carriers should incorporate this information. © 2001 Cancer Research Campaign http://www.bjcancer.com


*British Journal of Cancer* (2001) **84**(4), 478–481
© 2001 Cancer Research Campaign
doi: 10.1054/ bjoc.2000.1605, available online at http://www.idealibrary.com on IDEAL®

http://www.bjcancer.com

### The rate of the 6174delT founder Jewish mutation in *BRCA2* in patients with non-colonic gastrointestinal tract tumours in Israel

**A Figer[1], L Irmin,[1,3] R Geva,[1] D Flex[1], J Sulkes,[2] A Sulkes[1] and E Friedman[3]**

[1]Institute of Oncology and the [2]Epidemiology Unit, Rabin Medical Center (Belinson Campus), Petach Tikva; [3] the Susanne Levy Gertner Oncogenetics Unit, Chaim Sheba Medical Center, Tel-Hashomer, 52621, and the Sackler Faculty of Medicine, Tel-Aviv University, Tel-Aviv, Israel

**Summary** Inherited predisposition occurs in 5–10% of all gastrointestinal (GI) cancer patients, but with the exception of colorectal cancer (CRC), the genes involved in conferring genetic susceptibility remain largely unknown. Indirect evidence indicates that germline mutations in *BRCA2* might be associated with an increased risk for various GI malignancies. A single mutation (6174delT) occurs in the *BRCA2* gene in high-risk breast ovarian cancer families of Jewish Ashkenazi origin, in about 1% of the general Ashkenazi population, and rarely in non-Ashkenazi Jews. In order to assess the contribution of this germline mutation to non-CRC GI cancer in Jewish Israeli patients, we tested 70 unselected, consecutive Jewish Ashkenazi patients with gastrointestinal malignancies for this mutation by PCR amplification and modified restriction enzyme digests. Patients' age range was 38–90 years (mean 65.8±11.8 years). The most common malignancies were gastric cancer ($n = 35$) and exocrine pancreatic cancer ($n = 23$). Overall, 6 mutation carriers were detected: 3/23 (13%) of the patients with pancreatic cancer, 2/35 (5.7%) of patients with gastric cancer and 1/4 (25%) of patients with bile duct cancer. The 8.6% mutation carrier rate among patients is a rate significantly higher than that of the general Ashkenazi population (1.16% $P = 0.0002$). We conclude that the rate of the predominant Jewish *BRCA2* mutation in patients with gastric and pancreatic cancer significantly differ from that of the general population of the same ethnic origin. Thus, *BRCA2* mutations probably contribute to gastrointestinal tumorigenesis other then colon cancer, and the surveillance scheme for mutation carriers should incorporate this information. © 2001 Cancer Research Campaign http://www.bjcancer.com

**Keywords**: BRCA 2; Jewish founder mutation; non-colon cancer; inherited predisposition to cancer

Gastrointestinal malignancies other than colorectal cancer (CRC) are common. In Israel in 1995, gastric cancer was diagnosed in 572 Jewish individuals, pancreatic cancer in 368, 140 were diagnosed with primary liver or biliary tract cancer, and 110 had oesophageal cancer (Israel Cancer Registry, 1998). Furthermore, the mortality rate from gastric and pancreatic cancer is high: in Israel the age stratified rate (ASR) for gastric cancer mortality is 10.9/100 000 in Jewish men and 5.6/100 000 in Jewish women, compared with an ASR for morbidity of 14.2/100 000 and 7.7/100 000, respectively (Israel Cancer Registry, 1998). This high mortality rate stems in part from the paucity of early symptoms and hence the advanced stage at which these neoplasms are usually diagnosed. Thus, identifying individuals at high risk for developing these malignancies has obvious clinical implications. Familial clustering of cancer, a well known risk factor predisposing to gastric, pancreatic, and other GI cancer is noted in 3–10% of all incident cases of these malignancies (Zanghieri et al, 1990; Lynch et al, 1992; Fernandez et al, 1994). The relative risk for developing gastric cancer in first-degree relatives of gastric cancer patients ranges from 1.7 to 3.5, with an increase in relative risk associated with having more than one affected family member (Zanghieri et al, 1990; Palli et al, 1994; Lissowska et al, 1999). Similarly, familial clustering of pancreatic cancer is associated with an increased risk for developing these neoplasms in all first degree relatives (Ghadirian et al, 1991; Lynch et al, 1992). These observations suggest an inherited predisposition to these cancer types in a subset of patients, but the genes that underlie this genetic susceptibility remain largely unknown. Clustering of ovarian and gastric cancer (Easton et al, 1996) and breast/ovarian and exocrine pancreatic cancer (Tulinius et al, 1992) have been reported, suggesting a role for *BRCA2* gene mutations in pancreatic cancer predisposition (Phelan et al, 1996). Furthermore, a large study encompassing more than 3000 *BRCA2* mutation carriers and their first-degree relatives, estimated the relative risk (RR) for developing cancers other than breast/ovarian: for pancreatic cancer the RR was 3.51, for gallbladder and bile duct cancer – 4.97 and for stomach cancer – 2.59 (BCLC, 1999).

*Received 27 July 2000*
*Revised 2 November 2000*
*Accepted 8 November 2000*

*Correspondence to:* E Friedman

Among Jewish people, a single predominant mutation within the *BRCA2* gene (6174delT) occurs. This mutation can be detected in individuals at risk for developing breast and ovarian cancer (Abeliovich et al, 1997), in about 1–1.5% of the general Ashkenazi (East European) Jews (Ouddoux et al, 1996; Roa et al, 1996), and rarely among non-Ashkenazi Jews (Struewing et al, 1999). Analysis of 245 unselected patients with pancreatic cancer for this mutation, revealed two mutation carriers (0.8%) and an additional BRCA2 germline mutation carrier in a nearby codon (Goggins et al, 1996). However, not all patients in this latter study were Jewish individuals. Direct mutational analysis of 39 unselected Jewish Ashkenazi patients with pancreatic cancer, revealed 4 6174delT *BRCA2* (10%) mutation carriers (Ozcelik et al, 1997).

To test the notion that *BRCA2* mutations predispose to gastrointestinal malignancies other than colorectal cancer, we determined the rate of the *BRCA2* 6174delT predominant germline mutation in

70 unselected Jewish Ashkenazi patients with these types of malignancies who were consecutively treated in a single medical centre in Israel.

### MATERIALS AND METHODS

### Patients' characteristics and tumour material

All patients with a clinical and histopathological diagnosis of gastrointestinal malignancy (excluding colorectal cancer) who were treated at the Institute of Oncology, Rabin Medical Center from January 1, 1999 to March 31, 2000, were eligible for participation. The study was approved by the institutional review board, and all patients signed an informed consent. All consenting patients filled a detailed questionnaire that includes demographic data, past medical history, age at diagnosis, family history of cancer, especially gastrointestinal, breast and/or ovarian. Based on the criteria applied for other familial cancers, patients having at least one first-degree relative with GI or BRCA2 related cancer (breast and ovarian), or more than two second-degree relatives with cancer one of which is of GI, breast or ovarian origin, were classified as familial cases.

### DNA extraction

Anticoagulated peripheral blood was withdrawn by venopuncture, and DNA was extracted using standard techniques, using the Gentra kit (Gentra Inc., Minneapolis, MN).

### Mutation analysis of the predominant Jewish mutation in BRCA2

Mutational analyses for the predominant mutation (6174delT) in *BRCA2*, were carried out by restriction enzyme digest of amplified PCR products using modified amplification primers, to generate novel restriction sites, followed by restriction enzyme analysis to distinguish the mutant from the wild-type allele, as previously described (Rohlfs et al, 1997), and adopted by us (Bar Sade et al, 1998).

### Statistical analyses

Comparison of the rates of the founder Jewish mutation in *BRCA2* between the general Jewish Ashkenazi population (Hartage et al, 1999) and all GI cancer patients in our study group as well as the distribution within specific tumour types were performed using Fisher's Exact test. Odds ratio (OR) and the 95% confidence intervals (CI) were calculated from the tables

Even though the numbers for the Jewish Ashkenazi population are based on American Ashkenazi Jews (Hartage et al, 1999), we assumed that it is legitimate to use these numbers for two main reasons: first, the Ashkenazi Jewish population is well characterized as a distinct ethnic entity, regardless of the present place of residence (i.e. Tel-Aviv or Washington). Second, comparisons of Israeli and non-Israeli Ashkenazis with regard to being 185delAG *BRCA1* mutation carriers, did not show any differences between Israelis and Americans (Streuwing et al, 1997).

### RESULTS

### Patients' characteristics

All 70 were of Ashkenazi origin. The most common malignancy was gastric cancer ($n$ = 35), followed by exocrine pancreatic cancer ($n$ = 23), oesophageal cancer ($n$ = 7), bile duct cancer ($n$ = 4) and one small bowel cancer. Median age at diagnosis was 67 years (range 38–90 years) with a mean age of 65.8±11.8 years; 6 patients (8.57%) were diagnosed between 38–49 years; 16 (22.8%) – between 50–59 years; 19 (27.2%) – between 60–69 years; 20 (28.6%) – between 70–79 years; 9 (12.8%) over the age of 80 years. 13 (18.5%) and 12 (17.1%) patients had first-degree relatives with gastrointestinal or other cancer, respectively, and 4 (5.7%) had at least one first-degree relative with breast cancer.

**Table 1** Clinical and histopathological data of all *BRCA2* mutation carriers in the present study

| Pat no. | Sex | Tumour | Age at diagnosis | Family history of cancer |
|---|---|---|---|---|
| 1 | M | Bile duct cancer | 60 | Father – CRC<br>Son – CRC |
| 2 | F | Gastric | 55 | None |
| 3 | F | Gastric | 54 | Mother – BC<br>Grandmother – BC |
| 4 | M | Pancreas | 54 | Mother – CRC |
| 5 | M | Pancreas | 81 | None |
| 6 | M | Pancreas | 61 | Mother – abdominal cancer |

BC = Breast cancer; CRC = colorectal cancer.

### Germline mutational analysis

The presence of 6174delT *BRCA2* germline mutation was tested in all study participants and 6 carriers were found (6/70–8.6%). The clinical and pertinent data of the 6 mutation carriers are presented in Table 1. Notably, 3/23 of the patients with pancreatic cancer (13%), 2/35 (5.7%) of the patients with gastric cancer and 1/4 of the patients (25%) with bile duct cancer were mutation carriers. Surprisingly, only 1/5 individuals with a family history of breast and/or ovarian cancer was among the mutation carriers, and family history of other cancer was ascertained in 4/6 mutation carriers (Table 1). In addition, the age at diagnosis in mutation carriers was not noticeably younger than other individuals with the same cancer type.

### Statistical analyses

There was a statistically significant difference in the carrier rate of the 6174delT *BRCA2* mutation between the general Jewish Ashkenazi population and all cancer types in the present study

**Table 2** Comparison between the mutation carrier rate of the predominant Jewish mutation in *BRCA2* in the study population (Israeli Ashkenazi GI cancer patients) and the reference Jewish Ashkenazi population (from Hartage et al, 1999) by specific tumour types

| Cancer type | Mutation carriers | *P* value | OR | CI |
|---|---|---|---|---|
| Gastric | 2/35 (5.7%) | 0.06 | 5.2 | 1.2–22 |
| Pancreatic | 3/23 | 0.002 | 12.8 | 3.7–44.2 |
| Bile duct | 1/4 | 0.05 | 28.4 | 2.9–277.2 |
| Oesophageal | 0/7 | NS | – | – |
| Small intestine | 0/1 | NS | – | – |
| Total | 6/70 (8.6%) | 0.0002 | 8 | 3.3–19.2 |

NS – denotes statistically not significant.

© 2001 Cancer Research Campaign 

combined (59/5089 vs. 6/70, $P = 0.0002$). Analysis of the rate of this mutation within specific tumour types, showed that the rates in pancreatic cancer and bile duct cancer were also statistically significant higher than population controls (Table 2).

### DISCUSSION

In the present study, the involvement of the *BRCA2* gene in inherited predisposition to gastrointestinal cancer other than colorectal cancer was evaluated by direct mutational analysis. The rate of the predominant Jewish mutation in *BRCA2* in Ashkenazi patients with gastric, exocrine pancreatic cancer and bile duct cancer was significantly greater than the rate in the general Jewish Ashkenazi population (Struewing et al, 1997; Hartage et al, 1999). Furthermore, family history of cancer was elucidated in 4/6 mutation carriers, as an additional evidence that the mutation found is not merely an incidental finding, but rather reflects a true inherited predisposition.

The data presented herein are in agreement with other studies showing a higher than expected rate of *BRCA2* gene germline mutations in pancreatic cancer in ethnically diverse populations, and in Ashkenazi Jews, in particular. The original observation regarding finding of 2/245 (0.8%) 6174delT mutation carriers and 4/39 (10%) among unselected patients (Goggins et al, 1996) or Jewish individuals (Ozelick et al, 1997) with pancreatic cancer has been mentioned. Analysis of 38 Jewish individuals with pancreatic cancer, revealed 3 (7.9%) *BRCA2* 6174delT mutation carriers (Lal et al, 2000). Furthermore, in Iceland, where a single predominant mutation (999del5) in *BRCA2* exists, first-degree relatives of mutation carriers had a higher than expected rate of pancreatic cancer (Thorlacius et al, 1997). The role of *BRCA2* mutations in conferring genetic susceptibility to gastric, oesophageal, hepatic and biliary tract cancer is much less established. A high rate of allelic loss at the BRCA2 locus has been reported, but few somatic mutations have been detected in *BRCA2* in hepatocellular cancer (Katagiri et al, 1996). Similarly, 93% of oesopahgeal cancer from patients with a family history of the disease ($n = 23$), displayed allelic loss with a marker from the long arm of chromsome 13 (D13S894) (Hu et al, 1999). Moreover, in the most comprehensive study of cancer types other than breast and ovarian, associated with germline BRCA2 mutations, up to five-fold increased risk for biliary tract and more than double the risk for stomach cancer is reported (BCLC, 1999). However, to the best of our knowledge, no direct mutational analyses studies were ever performed in individuals with these latter cancer types.

The results of the present study which support other lines of indirect evidence as to the involvement of *BRCA2* germline mutations in the susceptibility to cancer of the upper gastrointestinal tract, should be reflected in genetic counselling. The increased risk for developing these malignancies should be incorporated into the routine counselling process, and surveillance schemes aimed at early detection of these cancer types should be devised and tested. Furthermore, the subset of Ashkenazi Jewish patients with either gastric, pancreatic cancer or bile duct cancer and a strong family history of cancer should probably all be tested for being *BRCA2* mutation carriers.

In conclusion, in Jewish individuals, germline mutations in the *BRCA2* gene seem to contribute to the genetic susceptibility to gastric, exocrine pancreatic and/or biliary tract cancer, and a family history of these or related cancer types. The predictive value, penetrance and the lifetime risk for developing these neoplasms in Jewish *BRCA2* mutation carriers remains to be determined in a larger, prospective study.

### ACKNOWLEDGEMENT

This study was funded in past by generous donation from Mr Ami Ya'ar in loving memory of his late wife, Ruthi.

### REFERENCES

Abeliovich D, Kaduri L, Lerer I, Weinberg N, Amir G, Sagi M, Zlotogora J, Heching N and Peretz T (1997) The founder mutation 185delAG and 5382insC in BRCA1 and 6174delT in BRCA2 appear in 60% of ovarian cancer and 30% of early-onset breast cancer among Ashkenazi women. *Am J Hum Genet* **60:** 505–514

Bar-Sade RB, Kruglikova A, Modan B, Gak E, Hirsh-Yechezkel G, Theodor L, Novikov I, Gershoni-Baruch R, Risel S, Papa MZ, Ben-Baruch G and Friedman E (1998) The 185delAG BRCA1 mutation originated before the dispersion of Jews in the diaspora and is not limited to Ashkenazim. *Hum Mol Genet* **7:** 801–805

Breast Cancer Linkage Consortium (1999) Cancer risks in BRCA2 mutation carriers. *J Natl Cancer Inst* **91:** 1310–1316

Easton DF, Matthews FE, Ford D, Swerdlow AJ and Peto J (1996) Cancer mortality in relatives of women with ovarian cancer: the OPCS Study. Office of Population Censuses and Surveys. *Int J Cancer* **65:** 284–294

Falk RT, Pickle LW, Fontham ET, Correa P and Fraumeni JF Jr (1988) Life-style risk factors for pancrcetic cancer in Louisiana: a case-control study. *Am J Epidemiol* **128:** 324–336

Fernandez E, La Vecchia C, D'Avanzo B, Negri E and Franceschi S (1994) Family history and the risk of liver, gallbladder, and pancrcatic cancer. *Cancer Epidemiol Biomarkers Prev* **3:** 209–212

Ghadirian P, Boyle P, Simard A, Baillargeon J, Maisonneuve P and Perret C (1991) Reported family aggregation of pancrcatic cancer within a population-based case-control study in the Francophone community in Montreal, Canada. *Int J Pancreatol* **10:** 183–196

Goggins M, Schutte M, Lu J, Moskaluk CA, Weinstein CL, Petersen GM, Yeo CJ, Jackson CE, Lynch HT, Hruban RH and Kern SE (1996) Germlinc BRCA2 gene mutations in patients with apparently sporadic pancreatic carcinomas. *Cancer Res* **56:** 5360–5364

Hartge P, Struewing JP, Wacholder S, Brody LC and Tucker MA (1999) The prevalence of common BRCA1 and BRCA2 mutations among Ashkenazi Jews. *Am J Hum Genet* **64:** 963–970

Hu N, Roth MJ, Emmert-Buck MR, Tang ZZ, Polymeropolous M, Wang QH, Goldstein AM, Han XY, Dawsey SM, Ding T, Giffen C and Taylor PR (1999) Allelic loss in esophageal squamous cell carcinoma patients with and without family history of upper gastrointestinal tract cancer. *Clin Cancer Res* **5:** 3476–3482

Israel Cancer Registry (1998) Cancer in Israel: facts and figures 1992–1995. Jerusalem

Katagiri T, Nakamura Y and Miki Y (1996) Mutations in the BRCA2 gene in hepatocellular carcinomas. *Cancer Res* **56:** 4575–4577

Lal G, Liu G, Schmocker B, Kaurah P, Ozcelik H, Narod SA, Redston M and Gallinger S (2000) Inherited predisposition to pancrcetic adenocarcinoma: role of family history and germ-line p16, BRCA1, and BRCA2 mutations. *Cancer Res* **60:** 409–416

Lissowska J, Groves FD, Sobin LH, Fraumeni JF, Jr; Nasierowska-Guttmejer A, Radziszewski J, Regula J, Hsing AW, Zatonski W, Blot WJ and Chow WH (1999) Family history and risk of stomach cancer in Warsaw, Poland. *Eur J Cancer Prev* **8:** 223–227

Lynch HT, Fasuro L and Lynch JF (1992) Familial pancreatic cancer. A family study. *Pancreas* **7:** 511–515

Odduoux C, Streuwing JP, Clayton CM, Neuhausen S, Brody LC, Kaback M, Haas B, Norton L, Borgen P, Jhanwar S, Goldgar D, Ostrer H and Offit K (1996) The carrier frequency of the BRCA2 6174delT mutation among Ashkenazi Jewish individuals is approximately 1%. *Nat Genet* **14:** 188–190

Ozcelik H, Schmocker B, Di Nicola N, Shi XH, Langer B, Moore M, Taylor BR, Narod SA, Darlington G, Andrulis IL, Gallinger S and Redston M (1997) Germline BRCA2 6174delT mutations in Ashkenazi Jewish pancreatic cancer patients. *Nat Genet* **16:** 17–18

Palli D, Galli M, Caporaso NE, Cipriani F, Decarli A, Saieva C, Fraumeni JF Jr and Buiatti E (1994) Family history and risk of stomach cancer in Italy. *Cancer Epidemiol Biomarkers Prev* **3:** 15–18

© 2001 Cancer Research Campaign

Phelan CM, Lancaster JM, Tonin P, Gumbs C, Cochran C, Carter R, Ghadirian P, Perret C, Moslchi R, Dion F, Faucher MC, Dole K, Karimi S, Foulkes W, Lounis H, Warner E, Goss P, Anderson D, Larsson C, Narod SA and Futreal PA (1996) Mutation analysis of the BRCA2 gene in 49 site-specific breast cancer families. *Nat Genet* **13:** 120–122

Roa BB, Boyd AA, Volick K and Richards CS (1996) Ashkenazi Jewish population frequencies for common mutations in BRCA1 and BRCA2. *Nat Genet* **14:** 185–187

Rohlfs EM, Learning WG, Friedman KJ, Couch FJ, Weber BL and Silverman LM (1997) Direct detection of mutations in the breast and ovarian cancer susceptibility gene BRCA1 by PCR-mediated site-directed mutagenesis. *Clin Chem* **43:** 24–29

Struewing JP, Hartge P, Wacholder S, Baker SM, Berlin M, McAdams M, Timmerman MM, Brody LC and Tucker MA (1997) The risk of cancer associated with specific mutations of BRCA1 and BRCA2 among Ashkenazi Jews. *N Engl J Med* **336:** 1401–1408

Struewing JP, Coriaty ZM, Ron E, Livoff A, Konichezky M, Cohen P, Resnick MB, Lifzchiz-Mcrecrl B, Lew S and Iscovich J (1999) Founder BRCA1/2 mutations among male patients with breast cancer in Israel. *Am J Hum Genet* **65:** 1800–1802

Thorlacius S, Sigurdsson S, Bjarnadottir H, Olafsdottir G, Jonasson JG, Tryggvadottir L, Tulinius H and Eyfjord JE (1997) Study of a single BRCA2 mutation with high carrier frequency in a small population. *Am J Hum Genet* **60:** 1079–1084

Tulinius H, Egilsson V, Olafsdottir GH and Sigvaldason H (1992) Risk of prostate, ovarian and endometrial cancer among relatives of women with breast cancer. *Br Med J* **305:** 855–857

Zanghieri G, Di Gregorio C, Sacchetti C, Fante R, Sassatelli R, Cannizzo G, Carriero A, Ponz de and Leon M (1990) Familial occurrence of gastric cancer in the 2-year experience of a population-based registry. *Cancer* **66:** 2047–2051

*© 2001 Cancer Research Campaign*
